# Morphological and Micromorphological Description of the Larvae of Two Endemic Species of *Duvalius* (Coleoptera, Carabidae, Trechini)

**DOI:** 10.3390/biology10070627

**Published:** 2021-07-06

**Authors:** Cristian Sitar, Lucian Barbu-Tudoran, Oana Teodora Moldovan

**Affiliations:** 1 Romanian Institute of Science and Technology, Saturn 24-26, 400504 Cluj-Napoca, Romania; 2 Zoological Museum, Babeș Bolyai University, Clinicilor 5, 400006 Cluj-Napoca, Romania; 3 Faculty of Biology and Geology, Babeș Bolyai University, Clinicilor 5, 400006 Cluj-Napoca, Romania; lucian.barbu@ubbcluj.ro; 4 INCDTIM Cluj-Napoca, Str. Donath 67-103, 400293 Cluj-Napoca, Romania; 5 Department of Cluj, Emil Racovita Institute of Speleology, Clinicilor 5, 400006 Cluj-Napoca, Romania

**Keywords:** *Duvalius* larvae, endemic species, Carpathians, sensilla, sensory complex, habitat

## Abstract

**Simple Summary:**

The *Duvalius* cave beetles have a wide distribution in the Palearctic region. They have distinct adaptations to life in soil and subterranean habitats. Our present study intends to extend the knowledge on the morphology of cave Carabidae by describing two larvae belonging to different species of *Duvalius* and the ultrastructural details with possible implications in taxonomy and ecology. These two species are endemic for limited areas in the northern and north-western Romanian Carpathians. Our study provides knowledge on the biology and ecology of the narrow endemic cave beetles and their larvae are important in conservation and to establish management measures. Endemic species are vulnerable to extinction and, at the same time, an important target of global conservation efforts.

**Abstract:**

The morphological and ultrastructural descriptions of the larvae of two cave species of Trechini—*Duvalius (Hungarotrechus) subterraneus* (L. Miller, 1868) and *Duvalius (Biharotrechus) paroecus* (J. Frivaldszkyi, 1865)—are presented in this paper. The interest in studying these larvae lays in their rarity and the limited distribution of the *Duvalius* species. The larvae were collected from caves in the Romanian Carpathians and were examined under a stereomicroscope and scanning electron microscopy. New important taxonomical and fine morphological characteristics are discussed together with conclusions on the larvae microhabitat as part of the measures to be taken by a proper management of caves.

## 1. Introduction

*Duvalius* (Delarouzée, 1859) species have distinct adaptations to life in soil and subterranean habitats, as do many species of the subtribe [1]. *Duvalius* has a wide distribution in the Palearctic region. Representatives of this genus were mentioned from Maghreb (Algeria), the western part of Europe in Spain (with some species in Catalonia and Mallorca) and France, central and southern Europe, Italy (especially in the Alps) but also Sicily and Sardinia, the Balkan Peninsula and the Carpathian Mountains. Towards the east, *Duvalius* is present from the Caucasus and the Middle East (Iran) to East China (Tien Shan Mountains) [2,3]. 

*Duvalius* consists of a complex [4] of troglomorphic genera [3], which were derived from local surface populations. Diverse ecological preferences and troglomorphism determined a speciose genus with 359 species [3,5], along with the large distribution range. Most species descriptions are based on the morphological description of the imago stage [6], and in the last decade have been completed by phylogenetic information based on molecular data [3]. 

The study of the biology of *Duvalius* representatives is difficult due to their hidden life, with the larvae even less studied than the adults [7]. Several contributions to the morphology of the preimaginal stages of some Carabidae in Europe have been published by Boldori [8,9] and on the subterranean larvae of the Trechini tribe by Jeannel [10], Giachino [11,12], Luff [13], Makarov and Koval [7], Casale and Marcia [14], Ortuño and Reboleira [15] and Giachino et al. [16]. The classic monographs of Jeannel [10,17], together with the work of Bousquet and Goulet [18], contributed substantially to the knowledge of the larval morphology of the Trechinae in general. Larval morphology of carabids was reviewed by Makarow [19].

However, there is little information about the larvae of subterranean Trechini species. The reproduction of cave adults and their larvae in the laboratory has been successful only in a few cases, due to the difficulty of recreating the cave’s relatively stable environmental conditions [14]. The general morphology of larvae within the Trechini tribe, with differences in chaetotaxy at the species level, is known [18,20,21]. Ultrastructural aspects have been studied by several authors for some Carabidae species: Altner and Bauer [22], Bauer and Kredler [23], Moore and Di Giulio [24] and Li et al. [25]. Giglio et al. [26] described the mouthparts sensilla for different Carabidae larvae. 

Our present study intends to extend the knowledge on the morphology of cave Carabidae by describing two larvae belonging to different species of *Duvalius* and the ultrastructural details, with possible implications in taxonomy and ecology. The larvae described here belong to *Duvalius (Hungarotrechus) subterraneus* (L. Miller, 1868) and *D. (Biharotrechus) paroecus* (J. Frivaldszkyi, 1865). These two species are endemic for limited areas, in the northern part of the Eastern Carpathians, respectively, and are vulnerable due to deforestation on the surface and non-organized tourism in caves. We will also discuss the habitats of the described larvae with remarks on the need for conservation measures in the caves.

## 2. Materials and Methods

### 2.1. Sampling Sites

**Figure 1:**
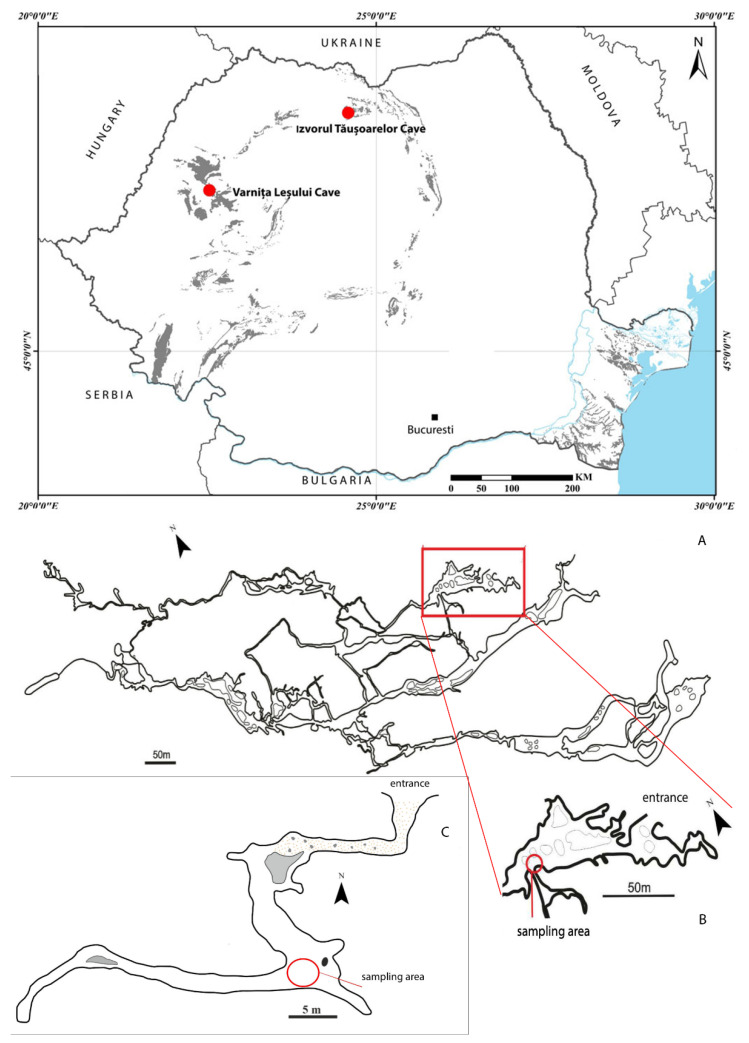
Map with the location of caves in Romania (upper) and the places inside the caves where the larvae were collected (**A**–**C**). (**A**,**B**) Izvorul Tăușoarelor Cave, redesigned and modified after Viehmann et al., (1964); (**C**) Varnița Leșului Cave, redesigned and modified after Viehmann et al. [29].

A larva of *Duvalius* was collected on 21 March 2019 (legit. C. Sitar, R. Năstase-Bucur) in the entrance zone of the Izvorul Tăușoarelor Cave (Rodna Mountains, northern Romania; Figure 1). The cave is located at 942 m a.s.l. and has a length of 8650 m, on a level difference of 329 m [27]. The habitat where the larva was collected is a descending slope with collapses and sediments, with dripping water, at about 100 m from the entrance. In the cave, adults of *D. (Hungarotrechus) subterraneus* (L. Miller, 1868) were also found and identified. The cave is protected under the Romanian legislation (O.U.G. nr. 57/2007) and considered as a scientific reservation with limited access.

In a passage of Varnița Leșului Cave (Pădurea Craiului Mountains, north-western Romania; Figure 1) a larva was collected on 19 January 1996 (legit. O. T. Moldovan). The cave is located at 520 m a.s.l. and has a development of 124 m [28]. The larva was found at about 100 m from the entrance, in the same place as adults of *D. (Biharotrechus) paroecus* (Frivaldszkyi, 1865), on the muddy cave floor, covered with humid degraded wood pieces. 

### 2.2. Microscopy Analysis

The specimens were examined under a stereomicroscope OLYMPUS SZX16 (OLYMPUS CORPORATION, Hamburg, Germany) and for details under an OLYMPUS BX51 (OLYMPUS CORPORATION, Hamburg, Germany) microscope. All drawings were prepared using camera lucida mounted on both microscopes. Measurements were made using a micrometer. The nomenclature and the location of pores and setae used here are according to those proposed by Bousquet and Goulet [18], Makarow [19] and Grebennikov and Maddison [20].

The two larvae were also prepared for scanning electron microscopy (SEM) using the turbomolecular pumped coater Quorum Q150T ES (Quorum Technologies, Laughton, UK), from the Integrated Electron Microscopy Laboratory (LIME) of the National Institute for Research and Development of Isotopic and Molecular Technologies (INCDTIM), Cluj-Napoca, Romania, and examined with an SEM Hitachi SU8230 (Hitachi Group, Tokyo, Japan) (LIME-INCDTIM, Cluj-Napoca, Romania). The preparation was carried out by Dr. Lucian Barbu.

## 3. Results

### 3.1. Description of Duvalius (Hungarotrechus) subterraneus (L. Miller, 1868) Larva

We assumed that this individual was a third instar larva based on the comparison with the data from the literature and with the larva described after.

#### 3.1.1. Habitus

**Figure 2:**
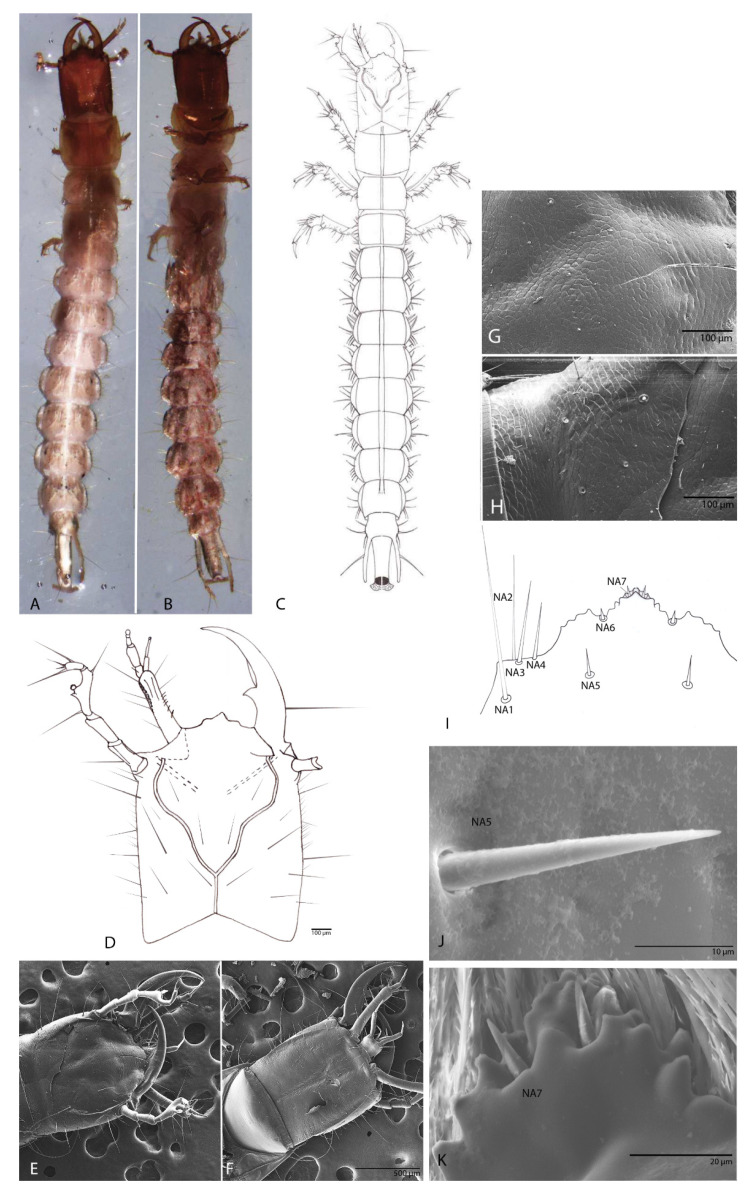
*Duvalius subterraneus*: (**A**) larva—dorsal view; (**B**) larva—ventral view; (**C**) schematic representation in dorsal view; (**D**) head dorsal view, schematic representation; (**E**) head features—SEM images—dorsal view; (**F**) head features—SEM images—ventral view; (**G**) microsculpture of the frontal sclerite; (**H**) microsculpture of the parietal sclerite; (**I**) nasale, schematic representation; nasale setae NA1–NA7; (**J**) SEM images of nasale setae NA5; (**K**) SEM images of nasale setae NA7.

Body slightly sclerotized, with setation. Body has an elongated shape; color brown-whitish. Body length (BL; from mandible to urogomphi apexes, macrosetae excluded) 8.3 mm (Figure 2A,B).

#### 3.1.2. Head

Longer than wide; flat; (length (HL): 1 mm; width (HW): 0.83 mm; ratio HL/HW: 1.20) anophthalmos; parallelized without the very slight constriction in the middle of the posterior lateral region as in other Trechinae; without postocular and epicranial grooves, any other structures (egg-bursters and teeth-like) are absent. Short epicranial suture, as long as one-fourth of the head width. Frontal sutures clearly visible, deeply curved and sinuous. Antennal ring strongly separated (Figure 2D,E). Microsculpture of the integument of the frontal and the parietal sclerite is clearly defined (Figure 2E,F). The frontal sclerite microsculpture is isodiametric (Figure 2G) and the microsculpture of the parietal sclerite is transverse (Figure 2H).

#### 3.1.3. The Nasale (Anterior Margin of Epistome)

Is tri-lobed and almost symmetrical; with one median tooth and another seven teeth on each side; median lobe largest and medially protruding, projecting forwards (Figure 2I). With four setae (NA 1–4) in the insertion area of maxilla on both sides of head and three pairs setae (NA 5–7), short and thick (Figure 2J,K).

#### 3.1.4. Chaetotaxy of the Cephalic Capsule

**Figure 3:**
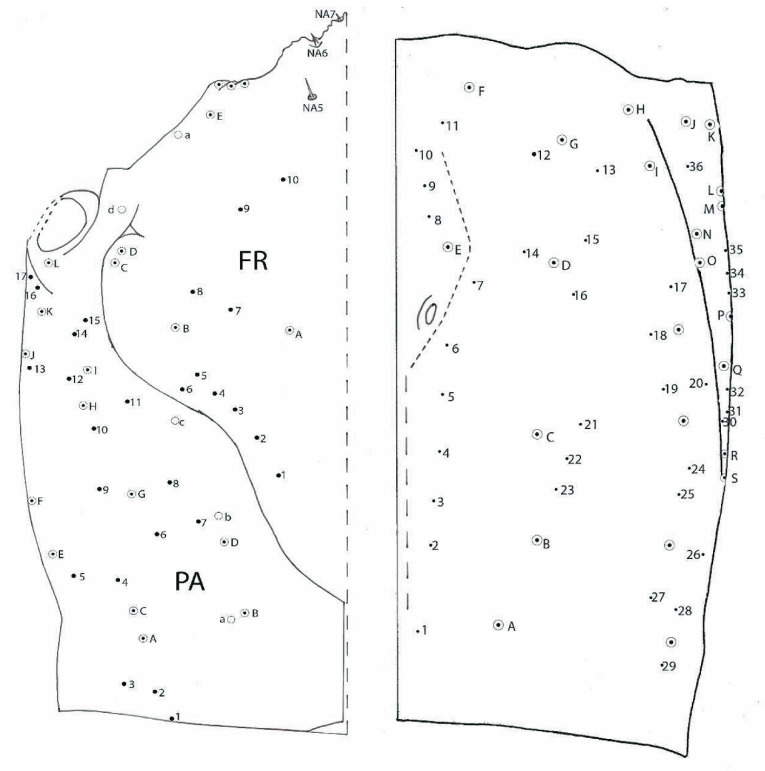
*Duvalius subterraneus*—cephalic capsule. Left-dorsal view: FR—frontal region, small setae 1–10, hypertrophied setae A–E, 1 pore a; PA—parietal region, small setae 1–17, hypertrophied setae A–L, pores a–d, nasale setae—NA5–NA7. Right-ventral view: small setae 1–36 and 17 hypertrophied setae A–Q; pores are absent.

Dorsally, on each side of frontal FR (Figure 3), 10 small setae (1–10), and 5 more hypertrophied setae (A–E) and 1 pore (a). Parietal PA with 17 setae (1–17), and 12 more hypertrophied setae (A–L) and 4 pores (a–d). Ventrally, on each side, 36 small setae (1–36) and 17 more hypertrophied setae (A–Q); pores are absent.

#### 3.1.5. Antenna

**Figure 4:**
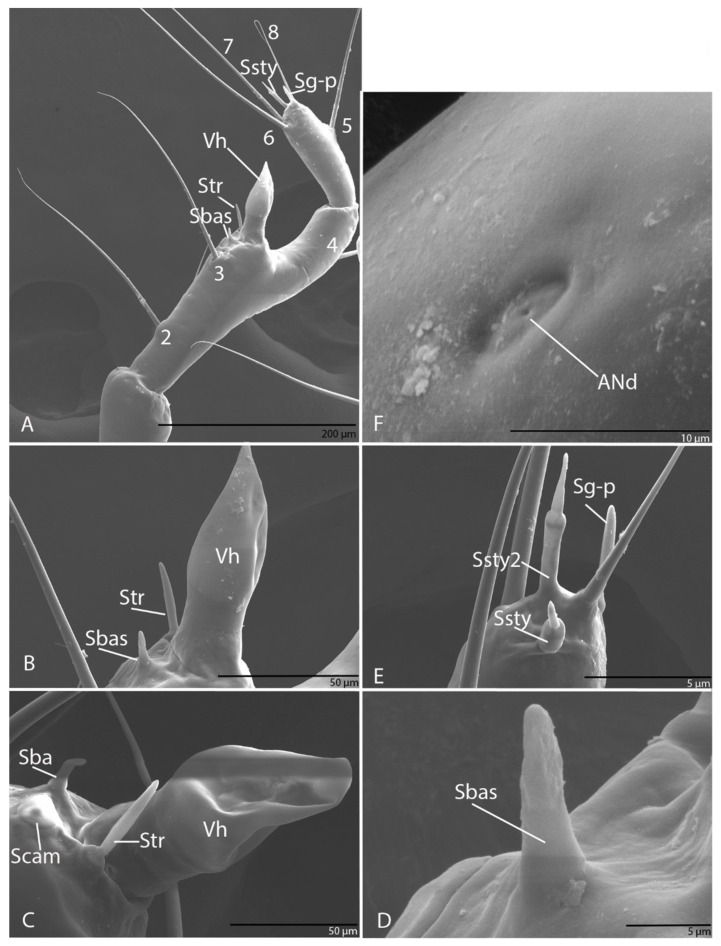
*Duvalius subterraneus*—antenna: (**A**) schematic representation; (**B**) ultrastructural details of sensilla complex—pores (ANa–ANd) and setae (AN1–AN8); (**C**) hyaline vesicle (Vh); (**D**) campaniform sensilla (Scam), trichoid sensilla (Str); (**E**) basiconic sensilla (Sbas); (**F**) styloconic sensilla (Ssty) and grooved peg sensilla (Sg-p).

Long, slender and with four segments; every segment longer than wide; first segment with three pores (ANa, ANb and ANc; Figure 4A); second segment with one seta (AN1; Figure 4A); third segment with three setae (AN2–AN4), long; on the apex of antennal segment, an obvious dome-shaped sensorial appendage, hyaline vesicle (Vh; Figure 4A–C); ultrastructurally, near Vh, one basiconic sensillum (Sbas) (Figure 4B–E), one trichoid sensillum (Str) and one campaniform sensillum (Scam) (Figure 4D,E). On fourth segment four setae (AN5–AN8) evident and very long, AN8 short (Figure 4A), one pore (Figure 4) and three sensorial appendages on the apexes: two styloconic sensilla (Ssty1–2) (Figure 4F) and one grooved peg sensillum (Sg-p) (Figure 4F).

#### 3.1.6. Mandible

**Figure 5:**
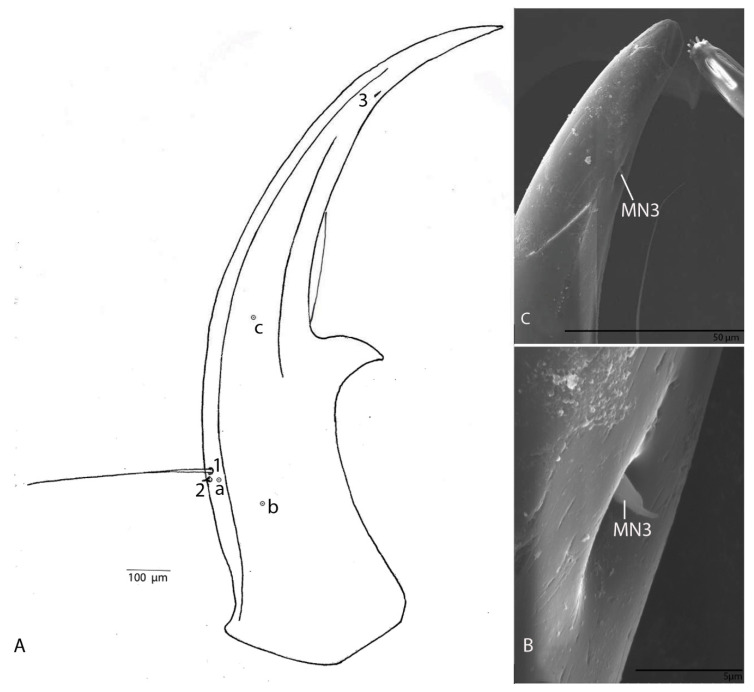
*Duvalius subterraneus*—mandible: (**A**) schematic representation—3 setae (MN1–MN3) and 3 pores (MNa–MNc); (**B**) ultrastructural details of mandible; (**C**) setae MN3 ultrastructural details.

Narrow, regularly and moderately curved, without additional teeth, only with a small excision in front of retinaculum (Figure 5A); with three setae (MN1–MN3) and three pores (MNa–MNc); MN1 is long, MN2 short and cylindrical, MN3 short and conical sensillum chaeticum; penicillum moderately developed (Figure 5B,C).

#### 3.1.7. Maxilla

**Figure 6:**
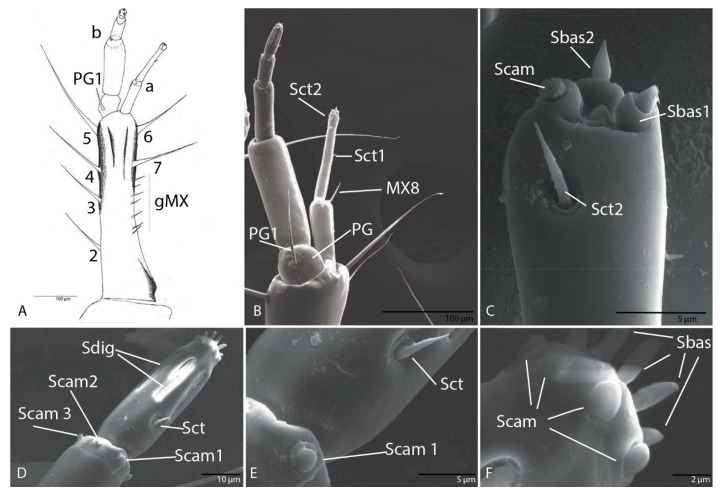
*Duvalius subterraneus*—maxilla: (**A**) schematic representation and (**B**) ultrastructural details of sensilla complex of maxilla: pores (MXa–MXc), setae (MX1–MX7 on the stipes and MX8 on first segment of galea), a variable number of short setae (gMX), palpiger (PG) with 1 seta (PG1), sensilla chaetica (Sct) 1–2 on first segment of galea; (**C**) second segment of galea—basiconic sensilla (Sbas), campaniform sensilla (Scam), sensilla chaetica (Sct2); (**D**) the fifth segment of maxillary palp—sensillum chaeticum (Sct), campaniform sensilla (Scam), digitiform sensilla (Sdig); (**E**) sensillum chaeticum (Sct), campaniform sensillum (Scam 1); (**F**) basiconic sensilla (Sbas) and campaniform sensilla (Scam) on the apex of the fifth segment of maxillary palp.

Cardo, very small with seta (MX1) not evident. Stipes with six setae (MX2–MX7) and a variable number of short setae (gMX). Lacinia absent. Galea with two segments (Figure 6A,B); second larger than the first; on first segment of galea one setae MX8 and one pore (MXa) (Figure 6A,B); second segment with two sensilla chaetica (Sct1–2) and three apical membranous sensilla, one campaniform sensillum (Scam) and two basiconic sensilla (Sbas1–2) (Figure 6B,C). Maxillary palps with five segments as in other representatives of the Trechini, composed of four palpomeres inserted on the basal segment—palpiger (PG) with one seta (PG1); second segment with one pore (MXb) larger than the others; on the third segment three sensilla chaetica and one pore (MXc) (Figure 6A); fourth segment with six campaniform sensilla (Scam 1–6) located at the distal end of the segment (Figure 6D); on the fifth one sensillum chaeticum (Sct) (Figure 6D,E), five digitiform sensilla with long pores (Figure 6D), and on the apex six campaniform sensilla, and six basiconic sensilla (Sbas) (Figure 6F).

#### 3.1.8. Labium

**Figure 7:**
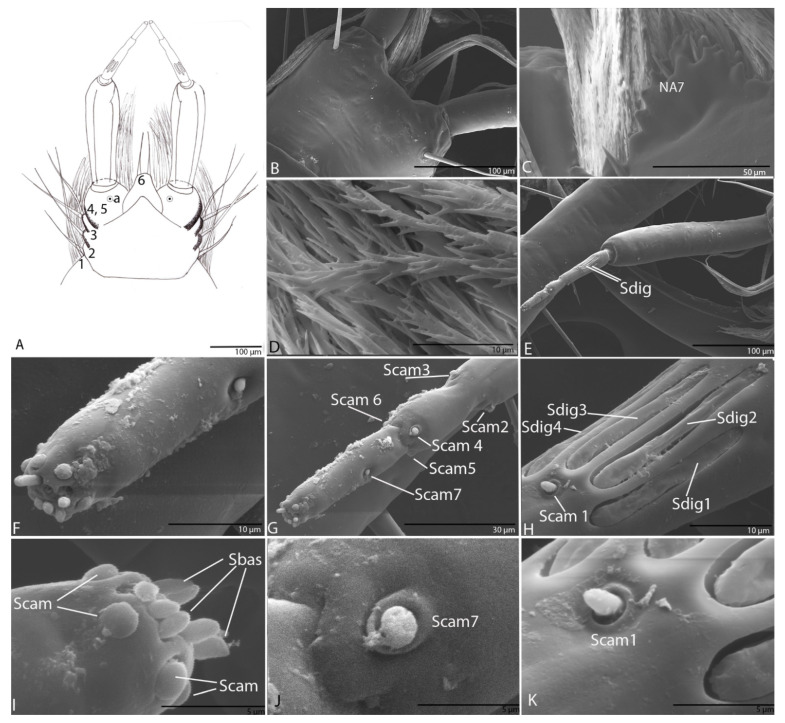
*Duvalius subterraneus*—labium: (**A**) schematic representation, prementum additional setae (1–6) and one pore (a); (**B**) ultrastructural details; (**C**) prementum located under the nasal is densely pubescent, nasal seta (NA7); (**D**) brushes with numerous digitiform diverticula; (**E**) digitiform sensilla (Sdig); (**F**) the apex of the third pseudo partition; (**G**) campaniform sensilla (Scam); (**H**) digitiform sensilla; (**I)** campaniform sensilla (Scam) and basiconic sensilla (Sbas) on the apex of the third pseudo partition; (**J**) campaniform sensilla (Scam 7); (**K**) campaniform sensillum (Scam 1).

Labial palps with two segments, as in other representatives of the Trechini, the second one subdivided into three apparent segments (Figure 7A,E,G). Prementum densely pubescent on the dorsal side (Figure 7C,D) and bearing six additional setae (LA1–LA6), five on the lateral margins and one pore (LAa) on each side (Figure 7A–D); the brushes have numerous digitiform diverticula along their entire length (Figure 7D); seta LA6 is half the length of the first segment of the palp. The second segment with a striated structure, a sensory complex according to Makarov and Koval (2003). On first pseudo partition surface with four digitiform sensilla (Sdig 1–4) with long pores (Figure 7E,H) and near Sdig3 one campaniform sensillum (Scam 1) (Figure 7H,K); at the end of the first pseudo partition two campaniform sensilla (Scam 2–3) (Figure 7G); on the second pseudo partition three campaniform sensilla (Scam 4–6) (Figure 7G); on the third pseudo partition, in its middle length, one campaniform sensillum (Scam 7) (Figure 7G,J) and on the apex six campaniform sensilla, and six basiconic sensilla (Sbas) (Figure 7F–I).

#### 3.1.9. Leg

**Figure 8:**
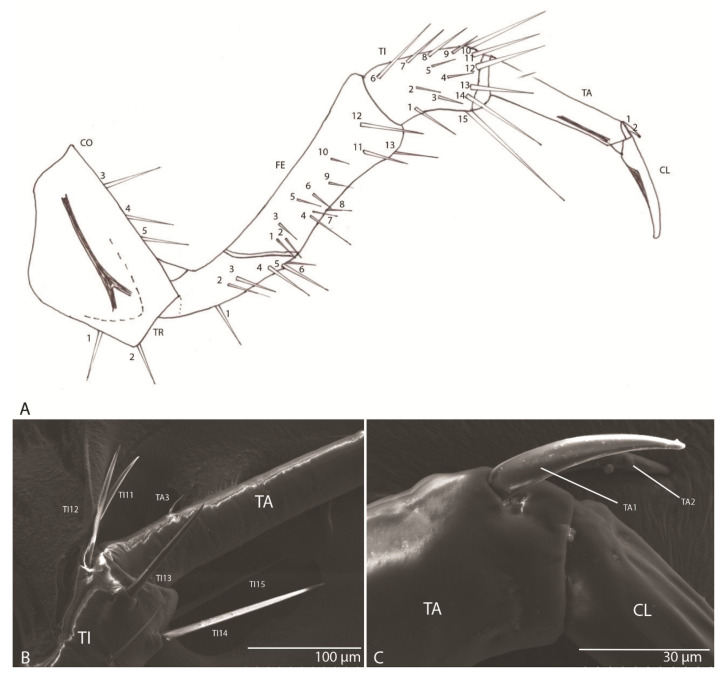
*Duvalius subterraneus*: leg—schematic representation and ultrastructural details. (**A**) leg segments and the setae corresponding to each segment which are marked with numbers; CO—coxa, 1-5 setae; TR—trochanter, 1-6 setae; FE—femur, 1-13 setae; TI—tibia, 1-15 setae; TA—tarsus, 1-2 setae; CL—claw. (**B**) setae TI10–TI14 on tibia; (**C**) setae TA1–TA2 on tarsus.

With one claw (Figure 8A): trochanter with six setae (TR1–TR6); femur with 13 setae (FE1–FE13); tibia with 15 setae (TI1–TI15) (Figure 8B); tarsus with three setae (TA1–TA3) (Figure 8C); slightly arched claw.

#### 3.1.10. Urogomphi

**Figure 9:**
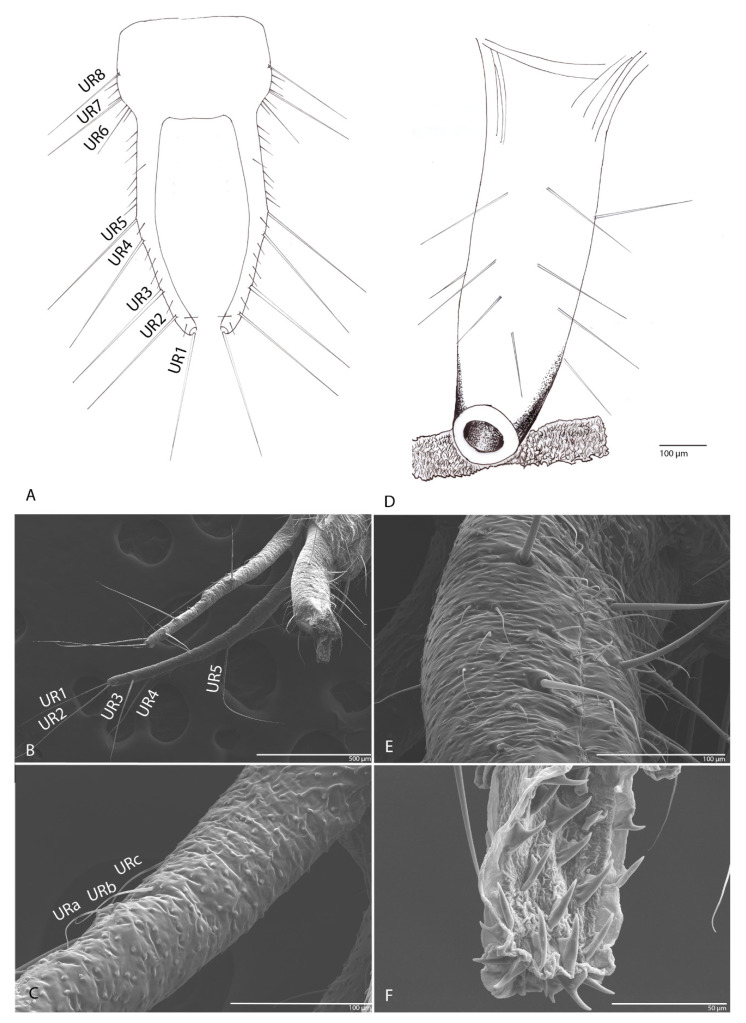
*Duvalius subterraneus*: (**A**) urogomphi—schematic representation, long setae (UR1–UR8); (**B**) urogomphi—ultrastructural details: setae UR1–UR5 are visible in the image; (**C**) sensilla chaetica (URa–URc) on urogomphi; (**D**) pygidium schematic representation; (**E**) pygidium ultrastructural details: 24 setae, skin with many rough surfaces; (**F**) retractable pseudopods in the anal tube with sclerotized teeth.

In dorsal view with eight long setae (UR1–UR8) on each side (Figure 9A,B); on the surface numerous sensilla chaetica (Figure 9C, URa–URc).

#### 3.1.11. Pygidium

With conical shape, 24 setae (Figure 9D,E) and the presence of two membranous structures, retractable pseudopods in the anal tube with sclerotized teeth (Figure 9F). The skin of both organs has many rough surfaces (Figure 9C,E).

### 3.2. Description of the Duvalius (Biharotrechus) paroecus (J. Frivaldszkyi, 1865) Larva

We assumed that this individual was a second instar larva based on the comparison with the data from the literature and with the larva described previously.

#### 3.2.1. Habitus

**Figure 10:**
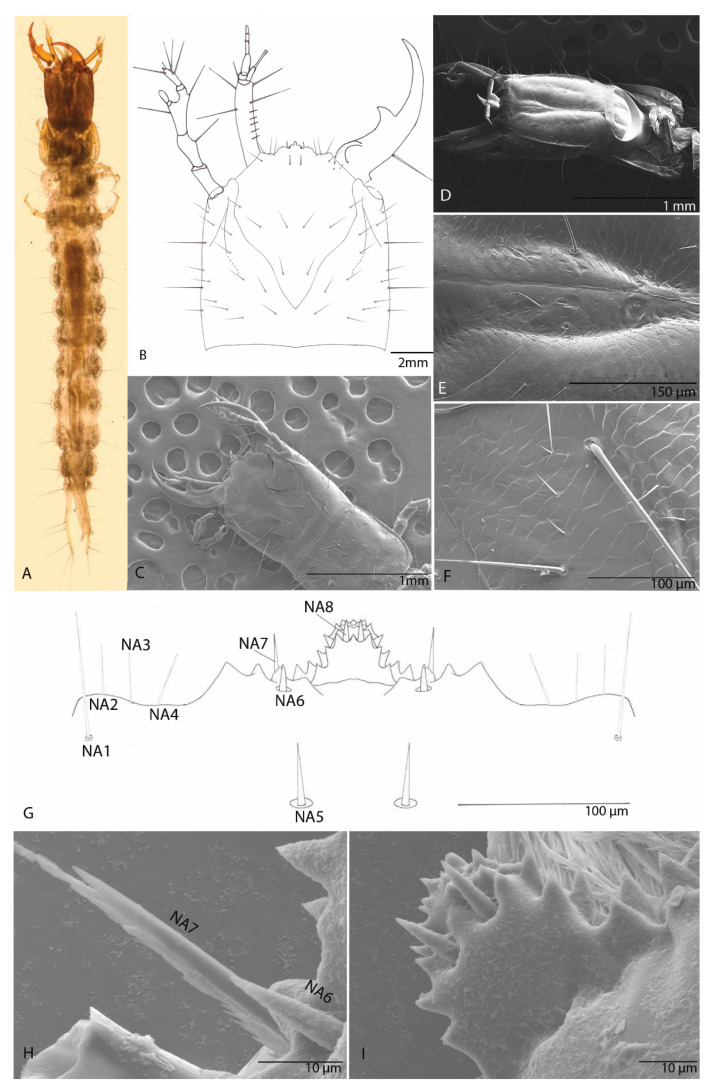
*Duvalius paroecus*: (**A**) larva—dorsal view; (**B**) head dorsal view, schematic representation; (**C**) larval head features—SEM images—dorsal view; (**D**) larval head features—SEM images—ventral view; (**E**) microsculpture of the ventral sclerite; (**F**) microsculpture of the frontal (FR) on dorsal side; (**G**) nasale, schematic representation; nasale setae NA1–NA8; (**H**) nasale—SEM images of nasale setae NA6 and NA7; (**I**) nasale—SEM images of nasale and setae NA8.

Body lightly sclerotized, with setation. Body has an elongated shape; color brown-whitish. Body length (BL; from mandible to urogomphi apexes, macrosetae excluded) 7.3 mm (Figure 10A).

#### 3.2.2. Head

It is as long as it is wide; flat; (HL: 0.8 mm; HW: 0.83 mm; ratio HL/HW: 1) anophthalmos; parallelized without a very slight constriction in the middle of the posterior lateral region as in other Trechinae; without postocular and epicranial grooves, any other structures (egg-bursters and teeth-like) are absent (Figure 10B). Short epicranial suture, as long as one-fourth of the head width. Frontal sutures clearly visible, deeply curved and sinuous. Antennal ring weakly separated (Figure 10B–D). Microsculpture of the integument of the frontal and the parietal sclerite is clearly defined. The frontal sclerite microsculpture is isodiametric, and the microsculpture of the parietal sclerite is transverse. On the ventral side, microsculpture of the integument respects the same distribution (Figure 10E,F).

#### 3.2.3. The Nasale (Anterior Margin of Epistome)

Is tri-lobed and almost symmetrical; median lobe largest and medially protruding, strongly projecting forwards, with two median teeth and another 11 teeth on each side; the teeth are arranged in layers (Figure 10G,I). The lateral lobes with four teeth on each of them. With four setae (NA1–4) in the insertion area of maxilla on both sides of head and four pairs of setae (NA 5–8), short and thick (Figure 10H,I).

#### 3.2.4. Chaetotaxy of Cephalic Capsule

**Figure 11:**
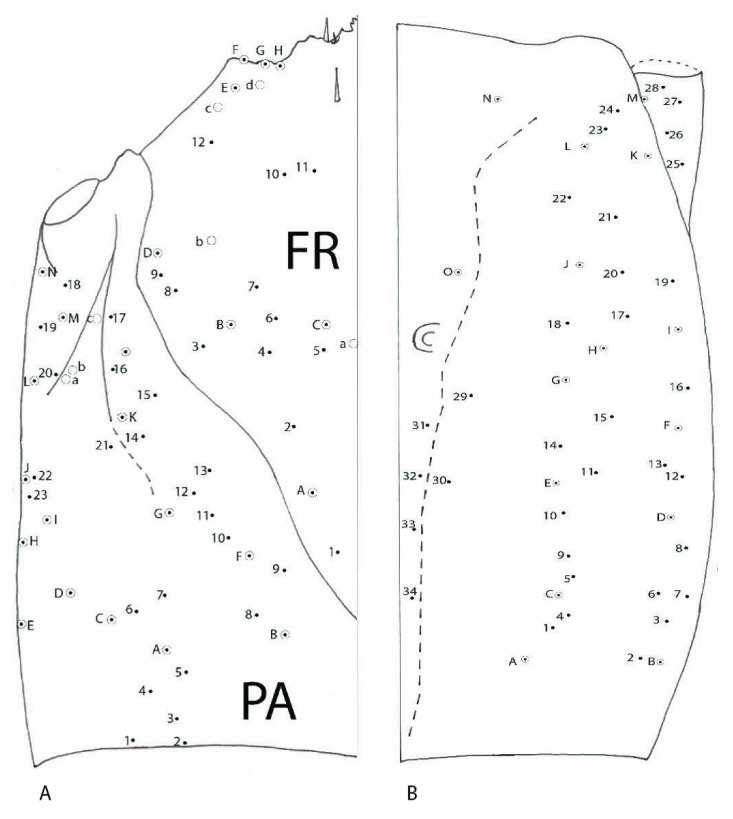
*Duvalius paroecus*—cephalic capsule: (**A**) dorsal view: FR—frontal region, small setae 1–12, hypertrophied setae A–E, 1 pore a; PA—parietal region, small setae 1–20, hypertrophied setae A–N, pores a–c; (**B**) ventral view: small setae 1–34 and 15 hypertrophied setae A–O, pores are absent.

Dorsal (Figure 11A)—on each side of frontal (FR) 12 small setae (FR1–FR12), in addition five more hypertrophied (FRA–FRE) and one pore a; parietal (PA) with 20 setae (PA1–PA20), in addition 14 more hypertrophied (PAA–PAN) and three pores a, b and c. Ventral (Figure 11B)—on each side 34 small setae (1–34); in addition, 15 more hypertrophied (A–O) pores are absent.

#### 3.2.5. Antenna

**Figure 12:**
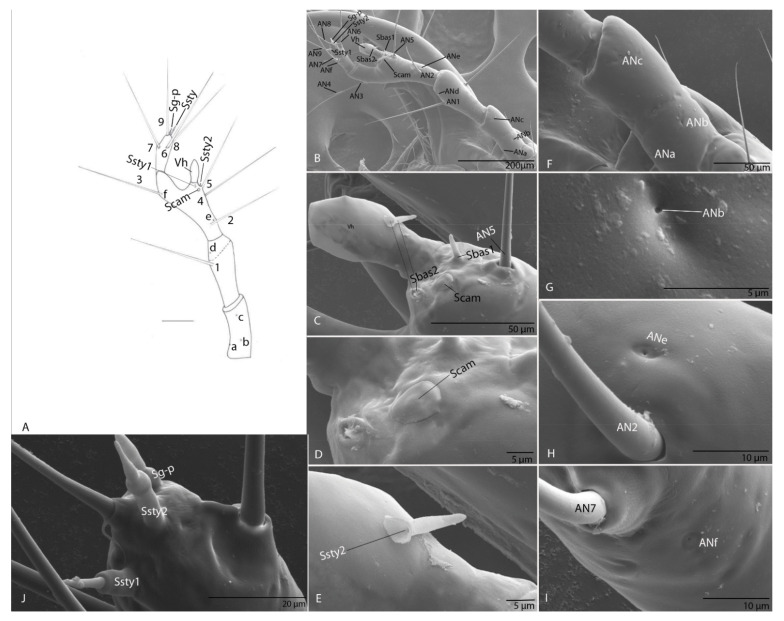
*Duvalius paroecus*—antenna: (**A**) schematic representation: setae 1–9, pores a–f, hyaline vesicle Vh, basiconic sensillum (Sbas), campaniform sensillum (Scam), styloconic sensilla (Ssty), grooved peg sensilla (Sg-p); (**B**) ultrastructural details (SEM): setae AN1–AN5, pores ANa–ANf, hyaline vesicle Vh, (**C**) basiconic sensilla (Sbas 1–2), campaniform sensillum (Scam), (**D**) campaniform sensillum (Scam); (**E**) styloconic sensillum (Ssty 2); (**F**) pores ANa–ANc; (**G**) pore ANb; (**H**) pore ANe; (**I**) pore ANf; (**J**) fourth segment of antenna with 3 sensorial appendages on the apexes: styloconic sensilla (Ssty1–2), grooved peg sensillum (Sg-p).

Long, slender and four segments; every segment longer than wide; first segment with three pores (ANa–ANc; Figure 12F,G); second segment with one seta (AN1) and one pore (ANd); third segment with four setae (AN2–AN5), long (Figure 12A,B), and two pores (ANe–ANf; Figure 12H,I); on the apex of antennal segment an obvious conic sensorial appendage—hyaline vesicle (Vh; Figure 12C) Ultrastructural, near Vh, two basiconic sensilla (Sbas1–2) and one campaniform sensillum (Scam) (Figure 12C–E). On fourth segment with four setae (AN6–AN9) evident and very long (Figure 12A), and three sensorial appendages on the apexes: two styloconic sensilla (Ssty1–2) and one grooved peg sensillum (Sg-p) (Figure 12J).

#### 3.2.6. Mandible

**Figure 13:**
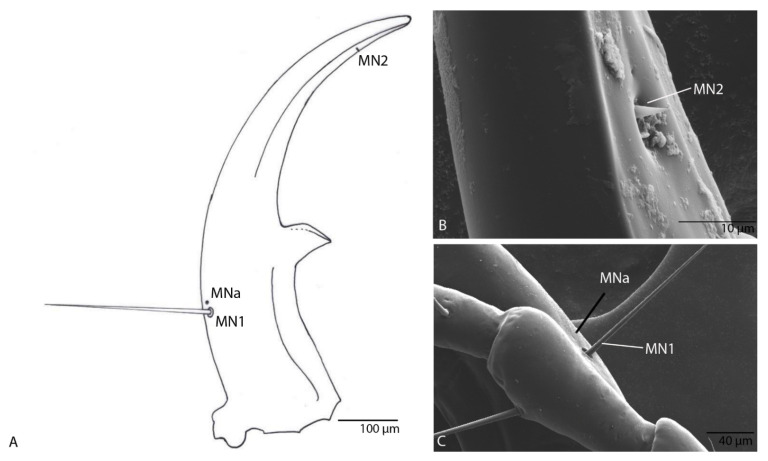
*Duvalius paroecus*—mandible: (**A**) schematic representation and ultrastructural details: 2 setae (MN1–MN2) and 1 pore (MNa); (**B**) SEM of por MNa and seta MN1; (**C**) SEM of seta MN2.

Narrow, regularly and moderately curved, without additional teeth, only with a small excision in front of retinaculum (Figure 13A); with two setae (MN1–MN2) and one pore (MNa; Figure 13B); MN1 is long, MN2 short and conical sensillum chaeticum as in the previous species (Figure 13C); penicillum moderately developed.

#### 3.2.7. Maxilla

**Figure 14:**
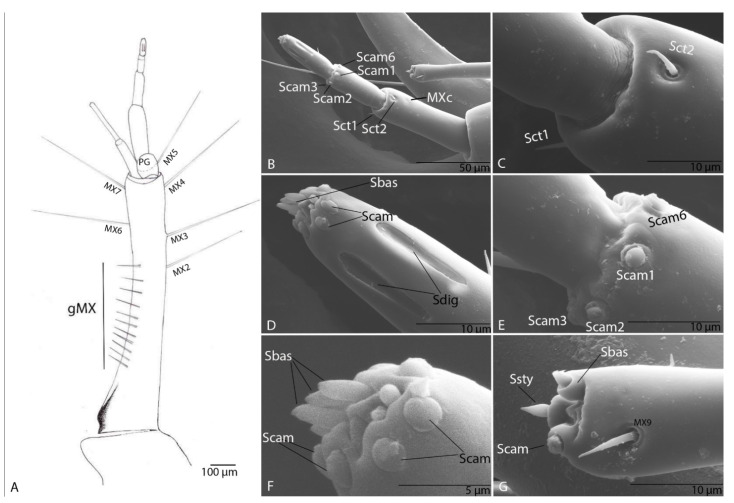
*Duvalius paroecus*—maxilla: (**A**) schematic representation: setae (MX1–MX7), a variable number of short setae (gMX), palpiger (PG) with 1 seta (PG1); (**B**) ultrastructural details of sensilla complex, sensilla chaetica (Sct1–2), pore MXc, campaniform sensilla (Scam1-3, Scam 6); (**C**) sensilla chaetica (Sct1–2) detail; (**D**) digitiform sensilla (Sdig), campaniform sensilla (Scam) and basiconic sensilla (Sbas) on distal end of the fifth segment; (**E**) campaniform sensilla (Scam1-3, Scam6) on distal end of the fourth segment; (**F**) campaniform sensilla (Scam) and basiconic sensilla (Sbas) on distal end of the fifth segment-detail; (**G**) distal end of the second segment of galea, seta MX9, campaniform sensillum (Scam), basiconic sensillum (Sbas), styloconic sensillum (Ssty).

Cardo very small with seta (MX1) not evident (Figure 14A). Stipes with six setae (MX2–MX7) and a variable number of short setae (gMX). Lacinia absent. Galea with two segments; second larger than the first; on first segment of galea one pore (MXa); second segment with two sensilla chaetica (MX8, MX9) and three apical membranous sensilla, one campaniform sensillum (Scam), one styloconic sensillum (Ssty) and one basiconic sensillum (Sbas) (Figure 14G). Maxillary palps with five segments as in others representatives of the tribe Trechini; it is composed by four palpomeres inserted on the basal segment—palpiger PG. On the third segment three sensilla chaetica (Sct 1–3) and one pore (MXc) (Figure 14B,C); fourth segment with six campaniform sensilla (Scam 1–6) located at the distal end of the segment (Figure 14B,E); on the fifth segment one sensillum chaeticum, three sensilla digitiformia (Sdig) with long pores, and on the apex and on the apex six campaniform sensilla, and six basiconic sensilla (Figure 14D,F) incompletely formatted. 

#### 3.2.8. Labium

**Figure 15:**
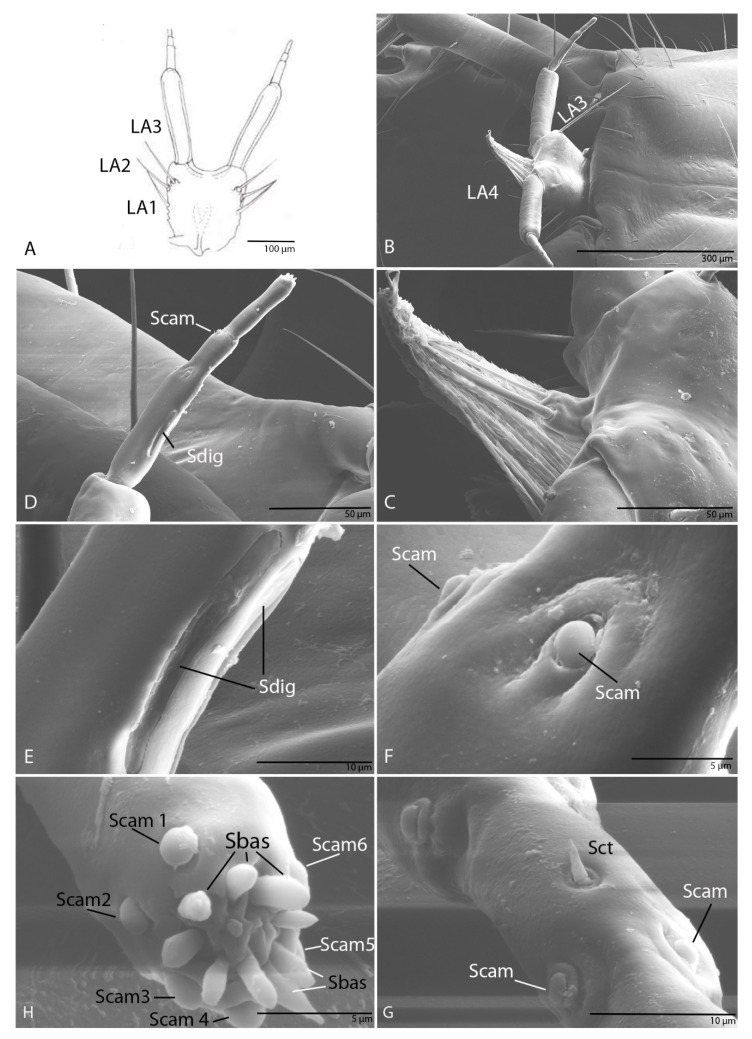
*Duvalius paroecus*—labium: (**A**) schematic representation, permentum setae LA1–LA3; (**B**) ultrastructural details, permentum setae LA3–LA4; (**C**) prementum densely pubescent; (**D**) the second segments of the labial palp, digitiform sensillum (Sdig), campaniform sensillum (Scam); (**E**) digitiform sensilla (Sdig); (**F**) campaniform sensilla (Scam) of the first pseudo partition; (**G**) campaniform sensilla (Scam) and sensillum chaeticum (Sct) of the second pseudo partition; (**H**) basiconic sensilla (Sbas) and campaniform sensilla (Scam 1–6) on the apex of the third pseudo partition.

Labial palps with two segments, as in other representatives of the tribe Trechini. The second segments subdivided into three apparent segments (Figure 15A,D). Prementum densely pubescent on the dorsal side and bearing four additional setae, three on the lateral margins (LA1–3; Figure 15A,B) on each side; the brushes have numerous digitiform diverticula as well as the species described above (Figure 15C). On first pseudo partition surface with two sensilla digitiformia with long pores (Figure 15D,E) and three campaniform sensilla (Figure 15D); on the second pseudo partition three campaniform sensilla and one sensillum chaeticum (Figure 15F,G); on the third pseudo partition, in the middle of the length one campaniform sensillum (Figure 15D) and on the apex six campaniform sensilla (Scam 1–6), and nine basiconic sensilla (Sbas1–9; Figure 15H).

#### 3.2.9. Leg

**Figure 16:**
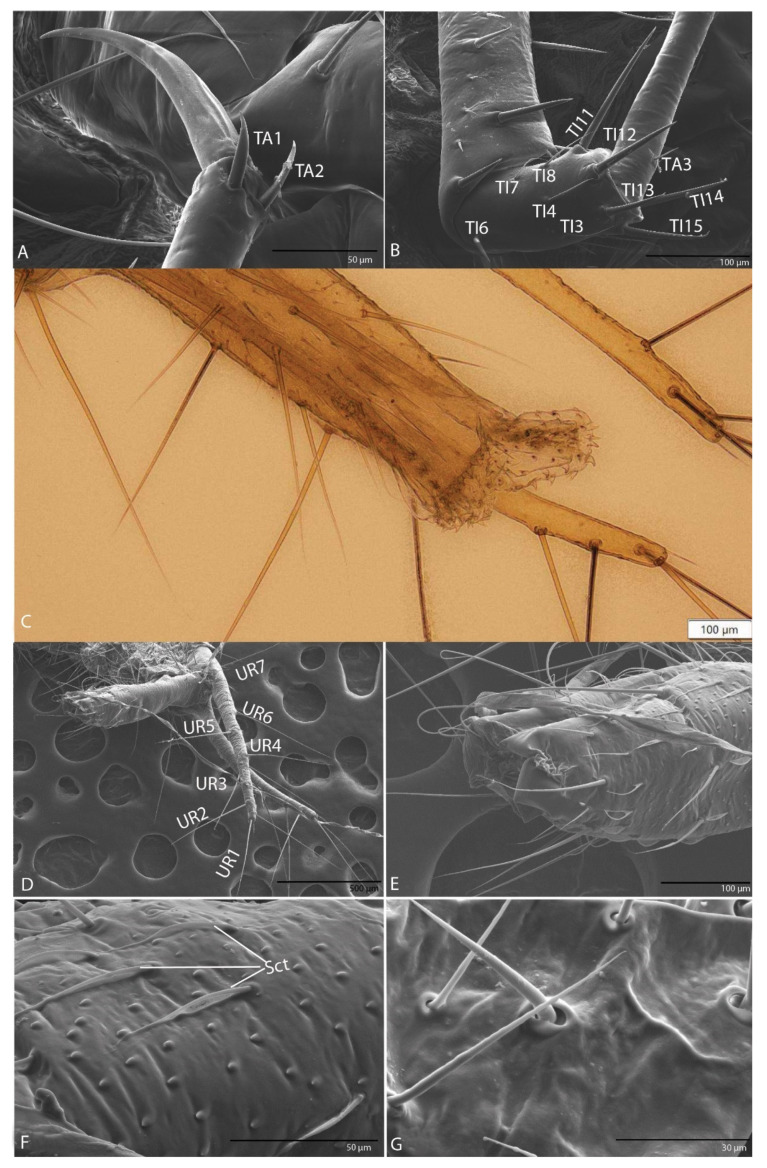
*Duvalius paroecus*: (**A**) leg ultrastructural details (SEM) of the claw and setae TA1–TA2; (**B**) leg ultrastructural details (SEM) of the tibia (TI) and setae (TI 4–15); (**C**) pygidium retractable pseudopods in the anal tube with sclerotized teeth; (**D**) urogomphi ultrastructural details: long setae (UR1–UR7); (**E**) pygidium ultrastructural details: retracted pseudopods in the anal tube; (**F**) sensilla chaetica (Sct) on urogomphi; (**G**) short setae on the anal tube.

With one claw (Figure 16A,B), similar to that of the previous species.

#### 3.2.10. Urogomphi

Is similar to that of the previous species (Figure 16C). In dorsal view, with eight long setae (UR1–UR8) on each side (Figure 16D); on the surface numerous sensilla chaetica (Sct) (Figure 16F).

#### 3.2.11. Pygidium

Is similar to that of the previous species (Figure 16C). With conical shape, 24 long setae, numerous short setae and two membranous retractable pseudopods in the anal tube with sclerotized teeth. The skin of both organs has many rough areas (Figure 16E–G).

## 4. Discussion and Conclusions

The larvae of these specialized troglobiotic (cave-adapted) carabid are difficult to find, even in the caves where adult individuals are abundant [10,30] (O.T.M., personal observations). Yet, another difficulty is to determine the presence or absence of sets or pores under an optical microscope for comparison with the classical description [2,15,18]. From our observations, the breaking of the setae at the time of collection and preparation can lead to confusion in identifying the pores and determining the number of setae under the optical microscope. Despite their importance, there are a few studies related to the ultrastructural organization of the head and appendages, as well as the sensilla present on these structures [22,23,24,25,26].

The larval morphology of the *Duvalius* larvae described in this paper conforms to the diagnosis of the Trechini by the absence of lacinia, presence of one claw, division of the apical palpal segments into apparent segments [7,13,18,31,32,33,34] and chaetotaxy [35]. The *Duvalius* larvae also have a set of apical and subapical sensilla on the antennae that are characteristic of the tribe [7], and the presence of secondary setae on the frontal part of the cephalic capsule [20]. The main morphological differences between the *Duvalius* larvae were considered to be the length/width ratio of the cephalic capsule, the shape of the nasale and slight differences in the development of setae [10]. These differences were also observed in the two described larvae, although it is not certain that they are individual variations and do not reflect inter-species variations. Therefore, we analyzed microstructures of *Duvalius* larvae, frequently overlooked due to the lack of SEM analysis, and that can provide more reliable taxonomic characters. The two larvae described here, *D. subterraneus* and *D. paroecus*, have a different chaetotaxy of the cephalic capsule (Figure 9 and Figure 11). These fine structures, such as nasale, number and arrangement of setae on the mandible or sensilla complex, represent structural elements of taxonomic importance and show the specialization of an organ such as the mandible, antenna and labium to subterranean microhabitats. The larvae are often found under rocks, in wet conditions, even inside the caves. The pubescence on the head and body, and the densely pubescent labium, shows possible adaptation to life on muddy soil and water film on a periodically flooded cave floor [14] or in “terrestrial-phreatic habitats” according to Jeannel [17]. The role of pubescence is to retain air bubbles in immersion conditions and to ensure floating [36]. The labial brushes observed in the two described larvae have multiple digitiform branches that increase the retention capacity of the organic particles from water [37], as also described in cave Leptodirini [38].

The nasale (2 I-K, 10 G–I) of the two species presents structural differences. The nasale of *D. subterraneus* is more prominent than of *D. paroecus. D. subterraneus* has seven setae and *D. paroecus* has eight. The nasale of the two species is tri-lobed (a central lobe and two lateral lobes). Each lobe has several small lobes at a fine structural level. These small lobes differ in number and shape between the two species. The central lobe of the two species is a formation consisting of two layers. The *D. subterraneus* first layer has four small lobes on each side. In the anterior part, between the two NA7 setae, the nasale has a slightly more prominent small lobe. The small lobes have a rounded apical part. The *D. paroecus* first layer has three small lobes on each side. The small lobes have a sharp apical part. The anterior small lobe (between the two NA8) is rounded and slightly contoured. The *D. subterraneus* second layer is an irregular formation in the anterior part. Laterally, there are three small, slightly contoured lobes on each side. The *D. paroecus* second layer is well contoured with five small sharp lobes on each side. In the anterior part, the second layer has six small sharp lobes.

Mandibles of the two larvae have a different number of setae (three for *D. subterraneus* (Figure 5A–C) and two for *D. paroecus* (Figure 13A–C). We noticed the presence of a chaetic sensillum on the inner side of the terebra at its distal third of each mandible (Figure 5A–C and Figure 13A–C). Until now, the presence of these sensilla has not been reported in the literature. The presence of this pair of sensilla in both species suggests that this character might be present in other *Duvalius* species, but was not observed under classical microscopes. The arrangement of the pairs of chaetic sensilla, both on the inner and on the outer curvature, highlights the possible mechanoreceptor role of these sensilla. Thus, in addition to the role in food intake, the mandibles provide information on the mandibles’ position relative to their food sources, and possible obstacles in the vicinity of the larva. Several authors have investigated the sensilla equipment of the larval head in Carabids: Altner and Bauer [22], Bauer and Kredler [23], Moore and Di Giulio [24], Li et al. [25] and Giglio et al. [26].

Attention was given especially to the ultrastructure and function of a sense organ located on the second segment. In carabid beetles, this structure is called the hyaline vesicle, and it was described by van Emden [31] and Jeannel [4]. It is a modified or complex sensilla basiconicum, very developed, and with an increased surface of the receptor membrane. This organ, with a presumed olfactory role, can receive highly diluted chemical cues [26,39]. On the last segment of the antenna there is a grooved peg sensillum, also mentioned by Altner and Prillinger [40], Keil [41], Steinbrecht [42] and Zacharuk [43], with an olfactory receptor or receptors with a combined olfactory/thermoreceptive function [40,44,45]. Two sensilla styloconica with possible thermo-hygroreceptive function were present in our larvae, as also described by Altner and Loftus [46], Steinbrecht and Kittmann [47] and Zacharuk [45]. In both *Duvalius* larvae described here, the hyaline vesicles and grooved peg sensillum were well represented, which indicated sensitivity to substances dissolved in the humid cave air or water. 

The description of *Duvalius* larvae at an ultrastructural level highlights the presence of particular sensory ultrastructures with a fundamental role in the reception and transmission of information from the environment [48]. As with most beetles, carabid larvae have a large number of sensory structures located on the labial and maxillary palps, as these appendages are directly involved in the detection and recognition of food or prey by means of both tactile and chemical receptors [26]. The distal segments of both maxillary and labial palps possess very similar sensory structural units, which are the sensilla campaniformia, sensilla chaetica, sensilla basiconica and sensilla digitiformia [26]. Sensilla digitiformia are very interesting receptors with roles in thermoreception, hygroreception and CO_2_ reception [49]. Although initially thought to be vibration receptors, used by larvae to determine terrain conformation [50], their number, shape and size may differ from species to species, which can be an adaptation to the used microhabitats, soil, microcaverns, caves, etc. Sensilla campaniformia and sensilla chaetica are mechanoreceptors. Sensilla basiconica have the role of contact chemoreceptors [26]. Trichoid sensilla present on galea and maxillary palps have an olfactory role [51]. Bland et al. [52] suggested that these sensilla are involved in the chemoreception of pheromones.

Our study should be completed by comprehensive phylogenetic analysis on *Duvalius*. It will, thus, provide valuable information on the specificity of vulnerable cave microhabitats, knowledge needed to establish proper management of caves. Knowledge on the biology and ecology of the narrowly endemic cave beetles and their larvae are important in conservation and to establish management measures. Endemic species are vulnerable to extinction and, at the same time, an important target of global conservation efforts [53,54] because they have specific climatic and environmental requirements and limited dispersal capacity [55,56,57,58,59]. Cave endemics are of particular note, for their very narrow distribution and low or no resilience to human impact, similarly to island faunas [60]. For the two caves where the new larvae were sampled, the main conservation measures are the restricted and controlled access of tourists, especially in the context of a changing climate. Increasing temperatures on the surface can have an impact on the distribution of subterranean Trechini, a group with sensitive representatives, by a shift in their preferred habitats, from more superficial to deeper habitats (bigger voids inside the karstic massifs, such as the caves) [61]. Therefore, the delimitation of paths inside caves with *Duvalius* or other cave species is absolutely obligatory, to avoid the microhabitats of their adults and larvae. On the surface, conservation measures must aim at conserving the forest habitats above the caves, ensuring a humid and cooler environment for the more superficial possible habitats of cave beetles and other cave species. 

## Data Availability

Not applicable.
